# cRGD inhibits vasculogenic mimicry formation by down-regulating uPA expression and reducing EMT in ovarian cancer

**DOI:** 10.18632/oncotarget.8079

**Published:** 2016-03-15

**Authors:** Jiao Tang, Jianguo Wang, Lin Fan, Xiaoxuan Li, Na Liu, Wanxian Luo, Jihui Wang, Yifeng Wang, Ying Wang

**Affiliations:** ^1^ Department of Obstetrics & Gynecology, Zhujiang Hospital, Southern Medical University, Guangzhou 510280, China; ^2^ Cancer Research Institute, School of Basic Medical Sciences, Southern Medical University, Guangzhou 510515, China; ^3^ Department of Reproductive Medicine Center, The Women and Children Hospital of Guangdong, Guangzhou Medical University, Guangzhou 511442, China

**Keywords:** vasculogenic mimicry, uPA, cRGD, epithelial-mesenchymal transition, ovarian cancer

## Abstract

Vasculogenic minicry (VM), an alternative blood supply modality except to endothelial cells-mediated vascular network, is a potential therapeutic target for ovarian cancer due to VM correlated with poor prognosis in ovarian cancer patients. Accelerated extracellular matrix (ECM) degradation is prerequisite for VM formation induced by epithelial-mesenchymal transition (EMT). Previous reports demonstrate uPA has ability to degrade ECM thereby promoting tumor angiogenesis. Also, exogenous cRGD sequence enables to modulate uPA expression, attenuate EMT and suppress endothelial-lined channels. Till now, the correlation of uPA and VM formation and the effect of exogenous cRGD on VM formation remain unknown. Herein, we validate uPA expression is positively correlated with VM formation in ovarian cancer tissues (90 cases) and ovarian cancer cells (SKOV-3, OVCAR-3 and A2780 cells). In particular, silencing uPA experiments show that down-regulated uPA causes notable decrease for the complete channels formed by SKOV-3 and OVCAR-3 cells. Mechanism study discloses uPA promotes VM formation by regulating AKT/mTOR/MMP-2/Laminin5γ2 signal pathway. The result demonstrates uPA may serve as therapeutic target of VM for ovarian cancer. Also, it is found exogenous cRGD enables to inhibit VM formation in ovarian cancer via not only down-regulating uPA expression but also reducing EMT. Exogenous cRGD may be a promising angiogenic inhibitor for ovarian cancer therapy due to its inhibiting effect on VM formation as well as endothelial cells-mediated vascular network.

## INTRODUCTION

Ovarian cancer, one of the fetal malignancies of the female reproductive system, has the highest mortality rate among all gynecological oncology [1]. It has been well known oxygen and nutrition provided by blood vessels are mainly responsible for tumor growth, development and metastasis. Jain et al. have proposed a theory about suppressing tumor growth *via* inhibiting its blood vessel formation. However, tumor neovascularization only composing of vascular endothelial cell has been challenged by vasculogenic mimicry (VM) [2]. VM is an alternative way to provide sufficient blood perfusion for highly malignant solid tumors, such as ovarian cancer [1, 3], breast cancer [4, 5] and hepatocellular cancer [6, 7]. Accumulating studies have indicated that VM is correlated with five-year survival, tumor staging and metastasis in ovarian cancer patients [8, 9], suggesting VM may be a potential therapeutic target for ovarian cancer. Therefore, it has significant implication to investigate the molecular mechanism of VM formation and seek for its related inhibitors thereby improving treatment outcome of ovarian cancer.

The unusual channel pattern of VM is composed of highly malignant tumor cells and extracellular matrix (ECM). Tumor cells involved in VM formation exhibit endothelial phenotypes of mesenchymal cells which is similar to process of Epithelial-mesenchymal transition (EMT) [6]. EMT is a dynamic biological process characterized by loss of epithelial feature and the acquisition of mesenchymal feature as well as a change in cellular morphology [10], and EMT subsequently improves the motility of tumor cells. More importantly, reports suggest that EMT-like tumor cells more easily form VM structure [11, 12] and regulators contributing to EMT have been verified to involve in VM formation [13, 14]. However, EMT-like tumor cells require to dissolve and pass through ECM before VM formation, which is not an ignored process of VM formation.

uPA, a serine protease with multiple function, is secreted by many cancer cells including ovarian cancer cells [15], and uPA enables to stimulate tumor cell proliferation and adhesion. It has been reported the high level of uPA indicates poor prognosis, and American society clinical oncology have appealed that uPA should act as risk assessment and a possible treatment target [16]. In particular, uPA can accelerate tumor metastasis and promote tumor angiogenesis by degrading ECM and basement membranes such as laminin, fibronectin and collagen, allowing cells to migrate [17, 18]. However, it is unknown whether uPA enables to promote VM formation and possibly serves as a therapeutic target of VM based on uPA function to ECM degradation.

Integrin α_v_β_3_ with high expression in neovascular endothelial cells and cancer cells can regulate cellular activities involved in angiogenesis. It's reported that RGD domain enables to inhibit tumor neovascularization dependent on endothelial cells by specific and competitive binding with integrin α_v_β_3_ [19]. Compared with linear RGD, Cyclic RGD (cRGD) has much more binding sites of integrin α_v_β_3_, thus cRGD presents promising therapeutic efficiency in angiogenesis induced by endothelial cells. Also, it is worth to note that endogenic RGD domain also enables to down-regulate uPA expression through high affinity with integrin α_v_β_3_ [20]. Moreover, intergrin α_v_β_3_ enables to promote interactions between cells and cells or ECM and plays pivotal role in invasion, migration and EMT process involving VM formation [21–23]. Accordingly, it is provoked our interest to explore whether exogenous cRGD can act as VM antagonist to suppress its formation by down-regulating uPA expression and attenuating EMT.

Herein, we first examine the correlation of uPA with VM in the level of ovarian cancer tissues and cells, and then attempt to validate its underlying molecular mechanism and estimate therapeutic potential of uPA. Most importantly, it is necessary to investigate whether exogenous cRGD has capacity to inhibit VM formation *via* down-regulating uPA expression and reducing EMT at protein level, gene level and biological characteristics. In this study, we aim to seek effective inhibitors for VM formation thereby further enriching therapeutic strategies for ovarian cancer.

## RESULTS

### VM and uPA expression are correlated with clinicopathological data

**Figure 1:**
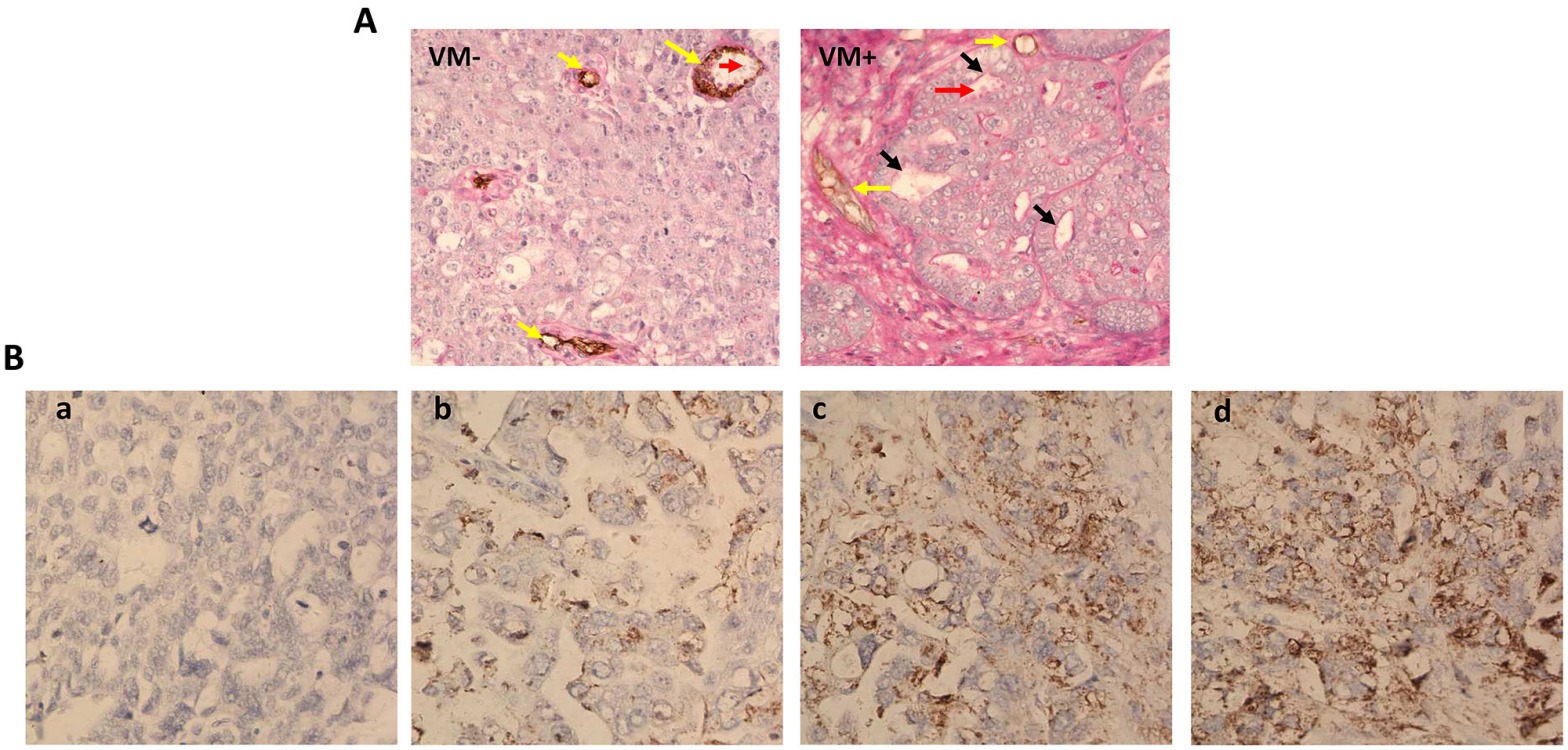
VM and uPA expression in ovarian cancer tissues **A.** In the VM (−) picture, endothelial cells were found by anti-CD34 immunohistochemistry staining, resulting in brown yellow product (represent with yellow arrows). VM channels lined by tumor cells and ECM were detected by CD34-PAS double staining in VM (+) picture (represent with black arrows) and even red blood cells could be detected in this channels (represent with red arrows). **B.** Different uPA expression levels were observed in ovarian cancer tissues by immunohistochemistry staining.

To determine whether VM could be discovered in tissues obtained from 90 ovarian cancer patients, we identified the endothelium and VM channel in ovarian cancer tissues using anti-CD34 and periodic acid-Schiff (PAS), respectively. CD34 positive represented that vessels formed by endothelial cells, while CD34 negative and PAS positive represented VM channels formed by tumor cells or ECM and even red blood cells could be found in these channels (Figure 1A). It was found 40 % tissues with VM channels were observed out of 90 ovarian cancer tissues, and then clinicopathological data of ovarian cancer tissues with VM (n=36) were compared with those without VM (n=54). In Table 1, we detected VM in 26 % of low FIGO stage and in 57.5 % of high FIGO stage as well as in 14 % G1, in 53 % G2 and in 64 % G3.

**Table 1:** Correlation between vasiculogenic mimicry, uPA and clinicopathplogical characteristic in ovarian cancer

Characteristics	VM				uPA		
	cases	Positive	negative	p-value	High(+++/++)	Low(+/−)	p-value
FIGO stage							
Low	50	13	37	0.003	18	32	0.000
High	40	23	17		33	7	
Histology							
Serous	63	29	34	0.076	38	25	0.167
Other	27	7	20		13	14	
Grade							
G1	36	5	31	.000	15	21	0.028
G2	32	17	15		19	13	
G3	22	14	8		17	5	
Age							
≤ 40	23	10	13	0.141	10	13	0.141
> 40	67	26	41		41	26	
uPA							
High	51	28	23	0.001			
Low	39	8	31				

uPA protein expression in ovarian cancer tissues was assessed by immunohistochemistry. We detected different expression of uPA in ovarian cancer tissues: the intensity of the dye color was graded as 0 (no color), 1 (light yellow), 2 (light brown), or 3 (brown), and the number of positive cells was graded as 0 (<5%), 1 (5-25%), 2 (25-50%), 3 (51-75%), or 4 (>75%). The two grades were added together and specimens were assigned to one of 4 levels: 0-1 score (−), 2 scores (+), 3-4 scores (++), more than 5 scores (+++). The positive expression rate was expressed as the percent of the addition of (++) and (+++) to the total number (Figure 1B). Then the correlation of uPA expression with clinicopathological data was analyzed in Table 1. High uPA expression (uPA ++ and uPA+++) was found in 36 % of low FIGO stage and in 82.5 % of high FIGO stage as well as in 41.6 % G1, in 59.4 % G2 and in 77.3 % G3. These results suggested VM and uPA expression had positive correlation with FIGO stage and Grade while no correlation with age and histology in ovarian cancer tissues which was consistent with previous reports [24].

### The correlation of uPA expression and VM in ovarian cancer tissues and cells

**Figure 2:**
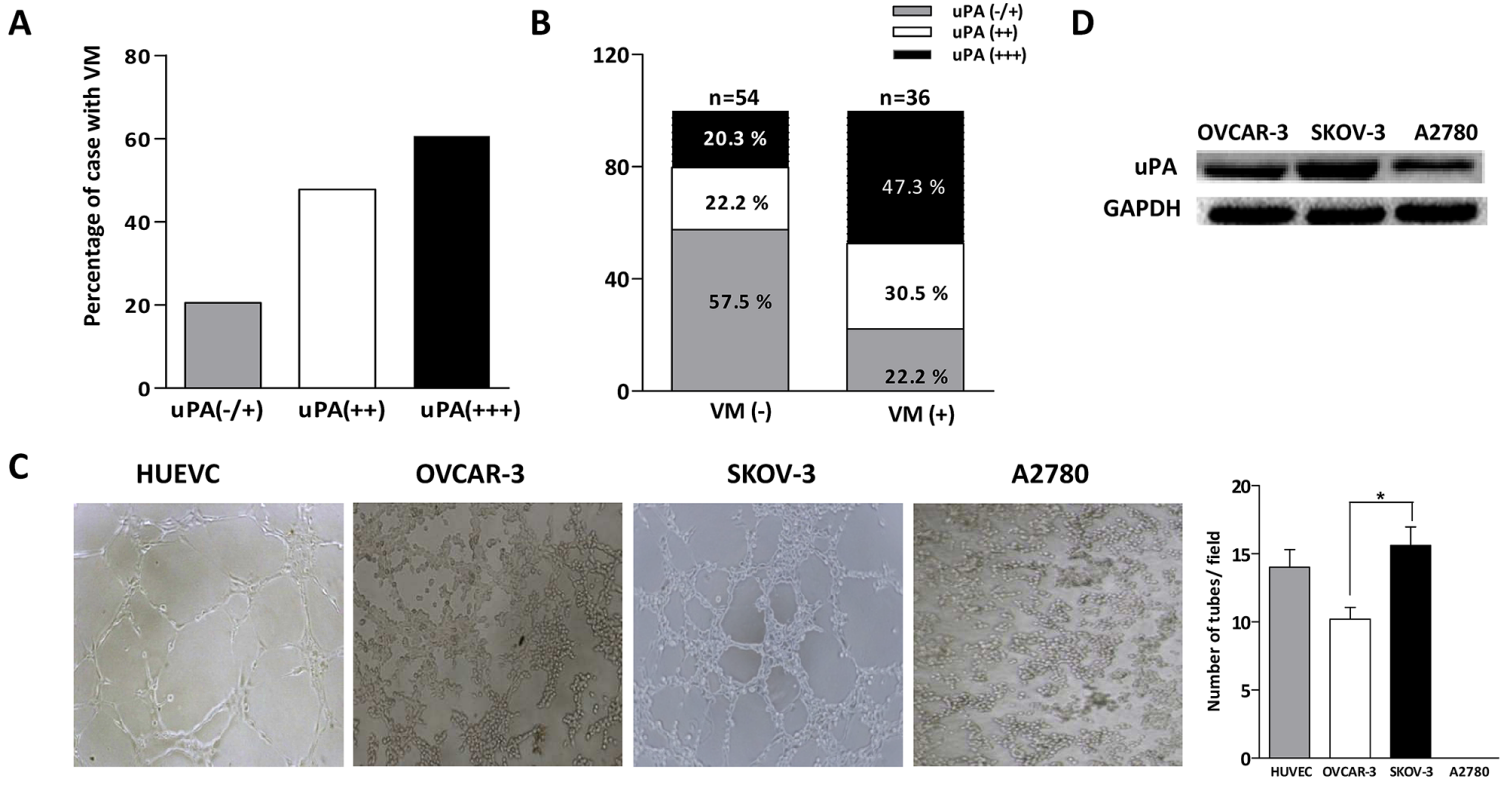
Correlation of uPA and VM formation in ovarian cancer tissues and cells **A.** Positive rate of VM increased in tissues with uPA (++) and uPA (+++) group compared with uPA (+/−) group. **B.** The share of high uPA expression (uPA+++) in the VM positive tissues was higher than in negative tissues. **C.** Western blot was performed to analyze uPA expression in OVCAR-3, SKOV-3 and A2780 cells. **D.** HUVEC, OVCAR-3, SKOV-3 and A2780 cells were cultured on Matrigel for 8 h (*P < 0.05).

To examine the correlation of uPA expression and VM, we analyzed uPA expression in tissues (VM+) and VM phenomenon in tissues with uPA expression. According to the level of uPA expression, all tissues were divided into three groups: uPA (−/+), uPA (++) and uPA (+++). It was observed more of VM in the uPA (+++) group: VM was detected in 18.1 % of uPA (−/+) tissues, 41.7 of uPA (++) tissues, and 57.1 % of uPA(+++) tissues (Figure 2A). Further analysis presented that uPA expression was up-regulated in VM (+) group: there were 22.2 % uPA (−/+), 30.5 % uPA (++) and 47.3 % uPA (+++), while there was only 20.3 % uPA (+++) in the VM (−) group (Figure 2B). These data indicated uPA expression was positively correlated with VM in ovarian cancer tissues.

We next validated the association of uPA and VM in ovarian cancer cells according to above results. Three-dimensional cultures were conducted to estimate the ability of the vessel-like channels formation in SKOV-3, OVCAR-3 and A2780 ovarian cancer cells using HUVEC cells as positive control. Cells were visualized by phase-control microscopy after incubation with 8 h. As shown in Figure 2C, we observed their different ability to form channels. It was found vessel-like channels formed by SKOV-3 and OVCAR-3 cells were similar to that of HUVEC cells, while the forming ability of SKOV-3 cells was better than that of OVCAR-3 cells. In contrast, no channels appeared in A2780 cells, even when the incubation time was prolonged to 24 h. Next, we investigated their expression level of uPA protein by western blot. As shown in Figure 2D, the expression of uPA protein in SKOV-3 cells was highest among them, and the expression of uPA protein in OVCAR-3 cells was higher than in A2780 cells. It was suggested that uPA expression had positive correlation with VM in ovarian cancer tissues and cells, and uPA might be involved in VM formation in ovarian cancer.

### The influence of down-regulated uPA on VM formation in ovarian cancer cells and its relevant molecular mechanism

**Figure 3:**
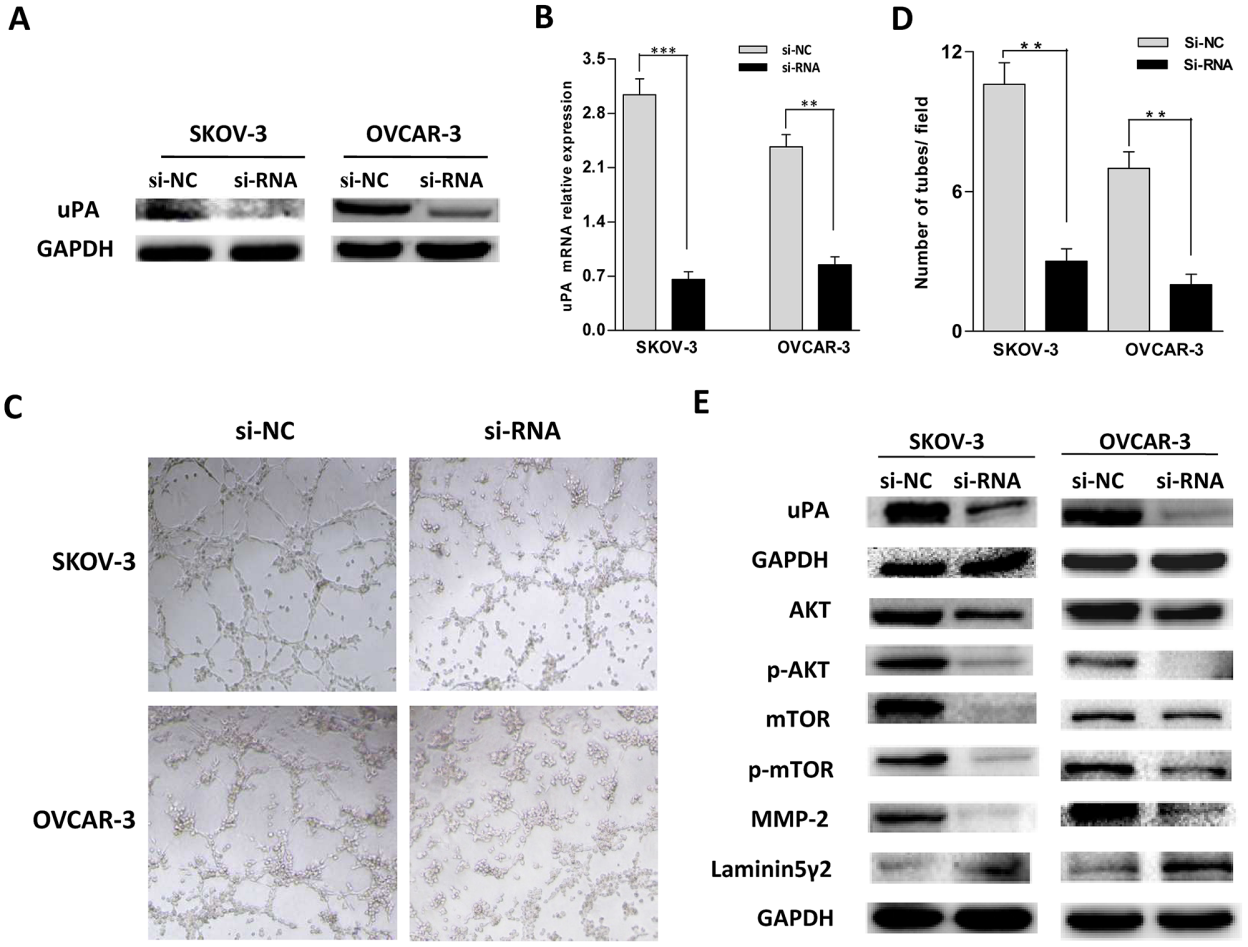
**A. B.** The interference effect of uPA-siRNA in SKOV-3 and OVCAR-3 cells detected by western blot and real time PCR (**P < 0.01, ***P < 0.001). **C, D.** SKOV-3 and OVCAR-3 cells subjected to different treatment were seeded on Matrigel for 8 h. **E.** Western blot analysis of uPA, AKT, p-AKT, mTOR, p-mTOR, MMP-2 and Laminin5γ2 in different treatment group in ovarian cancer cells.

Due to uPA function on ECM degradation and the correlation of uPA with VM, we then attempted to validate a hypothesis that uPA might participate in VM formation for ovarian cancer. We selected SKOV-3 and OVCAR-3 cells with high expression uPA to conduct the silencing uPA experiments. Specific uPA-siRNA fragments were transfected into both cells and the interfering effects of uPA were evaluated by RT-PCR and western blot. It was detected the expression levels of uPA mRNA and protein were obvious decreased after treating with uPA-siRNA (Figure 3A, B). In three-dimensional cultures, down-regulated uPA caused notable decrease for the complete channels formed by SKOV-3 and OVCAR-3 cells compared with their si-NC (Figure 3C,D). In summary, it was shown uPA might be a potential therapeutic target to inhibit VM formation in ovarian cancer.

It was reported that down-regulated uPA expression could inhibit AKT/mTOR signal pathway in glioblastoma cells [25], and AKT-mTOR signal pathway could modulate MMP-2 expression important for cleavage of the Laminin 5γ2 which was essential in the VM formation [26]. Thus, it might be speculated that uPA promoted VM formation via AKT/mTOR/MMP-2/Laminin5γ2. To verify it, the signal pathway of AKT/mTOR/MMP-2/Laminin5γ2 was detected in the interfered SKOV-3 and OVCAR-3 cells by western blot. As shown in Figure 3E, the expression of AKT, mTOR, their phosphorylation and MMP-2 was significantly decreased, while the uncleaved Laminin5γ2 was increased. It was confirmed that uPA promoted VM formation through AKT/mTOR/MMP-2/Laminin5γ2 signal pathway in ovarian cancer.

### The inhibiting effect of cRGD on VM formation in ovarian cancer cells and its relevant molecular mechanism

**Figure 4:**
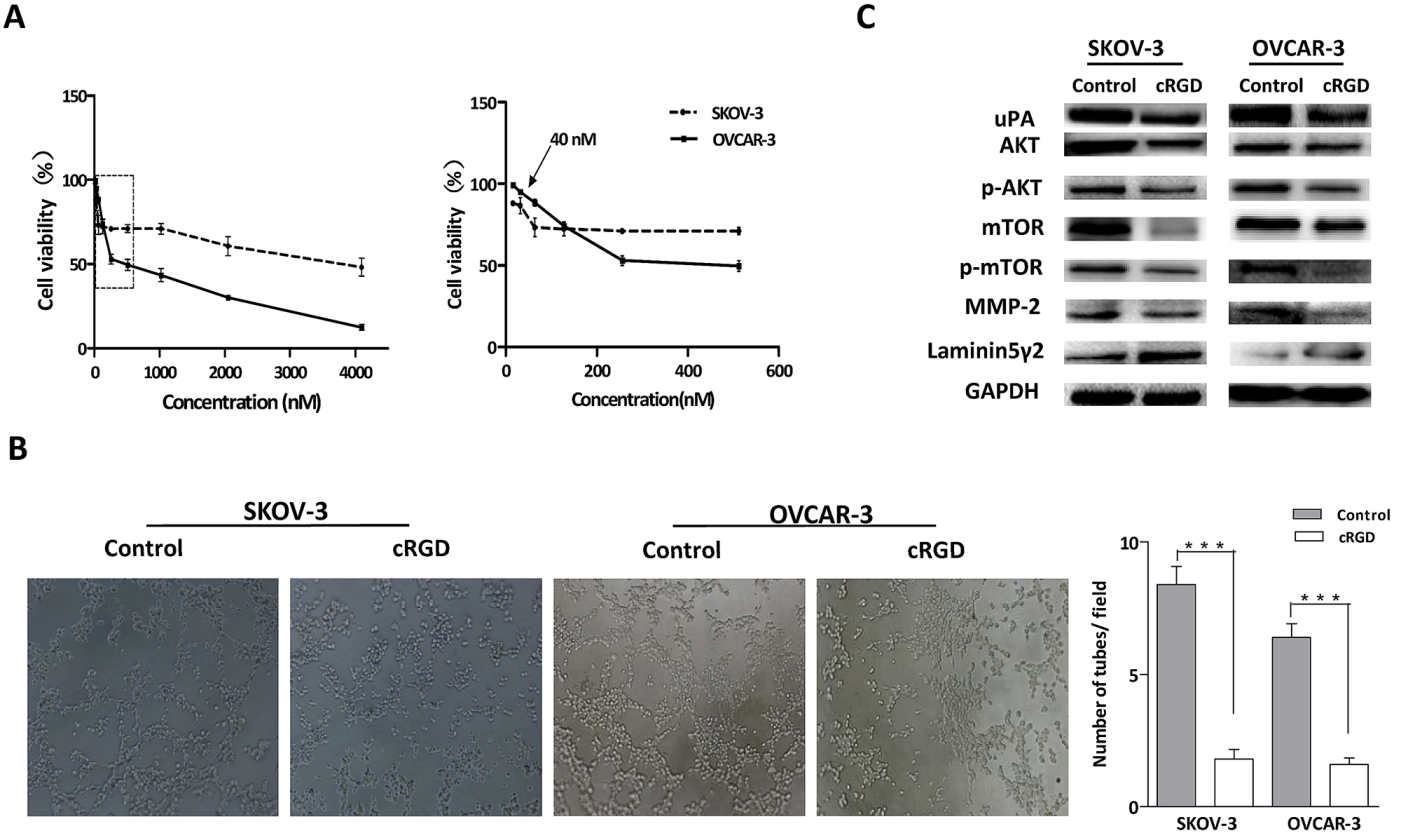
**A.** MTT assay was conducted to detect cytotoxicity of cRGD in ovarian cancer cells. **B, C.** SKOV-3 and OVCAR-3 cells treated with cRGD were cultured on Matrigel and their related protein were assayed by western blot (***P < 0.001).

According to above results, it was desirable to seek for uPA inhibitors and investigate their therapeutic potential for VM in ovarian cancer. Evidence demonstrated RGD domain could down-regulate uPA expression through specific binding to integrin αvβ3 [27]. Herein, we utilized cRGD as uPA antagonist to inhibit VM formation in ovarian cancer cells. To eliminate cRGD itself cytotoxicity, MTT assay was performed to screen appropriate cRGD dose (40 nM) far below their IC50 in SKOV-3 and OVCAR-3 cells (Figure 4A). Next, we estimated the inhibiting ability of cRGD to VM formation in three-dimensional cultures after SKOV-3 and OVCAR-3 cells were treated with cRGD, respectively. Compared with control groups, it was observed that cRGD could obviously suppress VM formation for both ovarian cancer cells (Figure 4B). uPA-mediated protein expression relevant to VM was evaluated by western blot in SKOV-3 and OVCAR-3 cells treated with cRGD for 48 h. We found that the changes of AKT/mTOR/MMP-2/Laminin5γ2 were similar as that of uPA interfernce experiments (Figure 4C).

**Figure 5:**
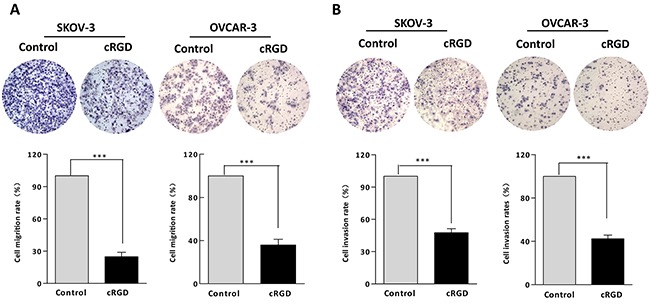
Migration and invasion experiments were conducted in **A.** SKOV-3 and **B.** OVCAR-3 cells treated with cRGD (***P < 0.001).

**Figure 6:**
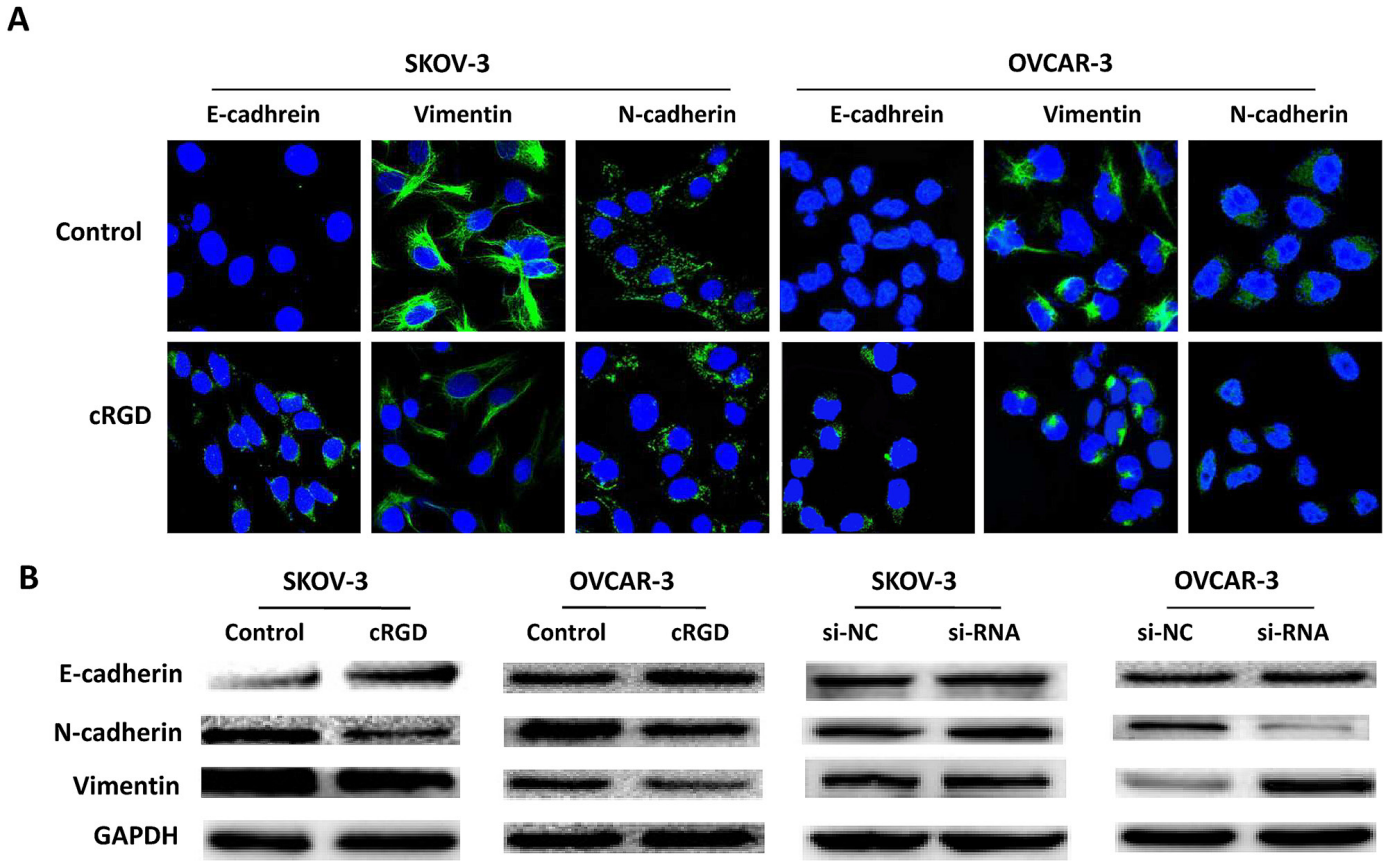
**A.** EMT-associated proteins were assessed by Immunofluorescence in SKOV-3 and OVCAR-3 cells after treating with cRGD or not. **B.** The expression of EMT-associated proteins (E-cadherin, N-cadherin and vimentin) were detected by western blot in SKOV-3 and OVCAR-3 cells after treating with cRGD or si-uPA.

**Figure 7:**
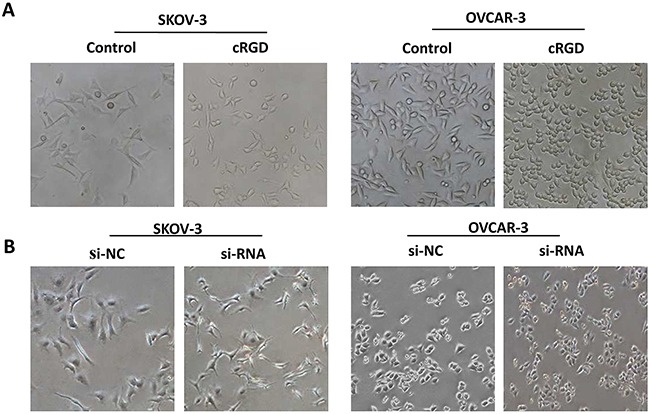
Phase-contrast images of SKOV-3 and OVCAR-3 cells **A.** after treating with cRGD or **B.** after treating with uPA si-RNA.

Due to the function of integrin α_v_β_3_ on EMT involved VM formation, we tested EMT characterization by assessing invasion and migration, and EMT-associated proteins in SKOV-3 and OVCAR-3 cells treated with cRGD. It was found the ability of migration and invasion for SKOV-3 and OVCAR-3 cells treated with cRGD was distinctly weakened compared with control groups (Figure 5A, B). We further assessed the expression of EMT-associated proteins by using immunofluoresence and western blot. It was found that cRGD increased the expression of epithelial marker (E-cadherin) and decreased the expression of mesenchymal marker (N-cadherin and Vimentin) in two cells by immunofluorescence, which was consistent with the results of western blot (Figure 6A,B). To investigate EMT reduction related with the decreased uPA, we conducted western blot to examine the changes of E-cadherin, N-cadherin and Vimentin in SKOV-3 and OVCAR-3 cells after down-regulating uPA (Figure 6B). There was no difference in these proteins for SKOV-3 cells treated with uPA si-RNA. As for OVCAR-3 cells, the silencing uPA expression led to the increased expression of Vimentin and no change for E-cadherin expression. According to the results, it indicated that the reducing EMT wasn't caused by the decreased uPA. Interestedly, N-cadherin expression for OVCAR-3 cells treated with uPA siRNA presented different trend which was inconsistent with the results of SKOV-3 cells. Accordingly, we compared the cellular morpology change after both cells treated with cRGD or uPA si-RNA, respectively. In observation of cell morphology, it was found that SKOV-3 and OVCAR-3 cells gained more cell polarity and more closely connected with the surrounding cells after cRGD exposure (Figure 7A). Compared with si-NC groups, they were no obvious difference in the si-RNA groups for SKOV-3 and OVCRA-3 cells (Figure 7B). It was reconfirmed the change of EMT was irrelevant to uPA for ovarian cancer. Taken together, cRGD had capability to suppress VM formation in ovarian cancer through down-regulating uPA expression and reducing EMT.

## DISCUSSION

Accumulating documents disclosed that tumor angiogenesis was dependent on not only endothelial cells-mediated vascular network but also VM formed by cancer cells and ECM [2, 28]. As an unusual way to supply blood for solid tumor, VM had important clinical significance for ovarian cancer, such as the positve correlation with poor prognosis, occurrence of distant metastasis and low survival rate [29–31]. Disclosure the molecular mechanism of VM had great implication for understanding ovarian cancer angiogenesis thereby providing a promising anti-angiogenic strategy. Evidences reported uPA protein was contributed to tumors development, and particularly uPA could promote tumor angiogenesis *via* degrading ECM and basement membranes [32]. In this study, we found positive correlation of uPA and VM formation in ovarian cancer and first verified uPA had ability to accelerate VM formation in ovarian cancer.

We found that uPA expression and VM had correlation with ovarian cancer grad and stage: the higher the tumor grade/stage, the higher the percentage of VM structures or uPA expression, which were in agreement with previous reports [9, 15]. And further analysis exhibited that the percentage of VM in uPA(+++) group was higher compared with uPA(−/+) or uPA(++) group, and the expression of uPA in VM (+) group was much higher than in VM (−) group. It suggested that uPA was positively associated with VM formation in ovarian cancer tissues. Several reports about uPA expression level in ovarian cancer cells were inconsistent. An Li et al. suggested that uPA expression in SKOV-3 cells was approximately similar in OVCAR-3 cells by immunostaining [24], while other study showed that uPA expression in SKOV-3 cells was higher than in OVCAR-3 cells by western blot [33]. In this study, we found that uPA expression in SKOV-3 cells was the highest among three ovarian cancer cells (SKOV-3>OVCAR-3>A2780 cells). And three-dimensional cultures presented that SKOV-3 and OVCAR-3 cells with higher levels of uPA were more prone to form vessel-like channels, compared with A2780 cells. It was indicated that uPA possibly correlated with VM formation in ovarian cancer cells. More importantly, we observed that the silence of uPA significantly reduced the formation of vessel-like channels in SKOV-3 and OVCAR-3 cells, further confirming positive contribution of uPA to VM formation in ovarian cancer.

Previous studies indicated that down-regulation of uPA led to little differences on the amounts of total PI3k and AkT whereas significantly less phosphorylated PI3k and Akt in glioblastoma cells [34]. Gondi CS et al. also found that the down-regulated uPA inhibited the PI3k/AkT pathway [25]. In addition, it was documented that the activation of PI3K/AKT/mTOR played an important role in invasion and migration, the mechanism of how this occured appeared to be through regulating MMPs [35]. Also, the molecular mechanism of VM was mainly focused on investigating VE-cadherin, EphA2 and MMPs [36–38]. MMPs enabled to divide Laminin5γ2 into γ2' and γ2x fragments which was an essential process for VM formation, and importantly, MMPs-Laminin5γ2 was the last stage in VM formation signal pathways [37]. In addition, Anil et al. reported MMP-2 participated in VM formation in ovarian cancer [39]. Therefore, MMP-2 and Laminin5γ2 enabled to act as evaluation proteins to assess VM formation in ovarian cancer. In this study, we found that the reduced uPA significantly decreased the levels of AKT, mTOR, their phosphorylation and MMP-2, and increased the level of uncleaved Laminin5γ2 in SKOV-3 and OVCAR-3 cells. All of these results suggested that uPA was a positive mediator for VM formation in ovarian cancer *via* AKT/mTOR/MMP-2/Laminin5γ2 signal pathway.

Solid tumor couldn't grow larger than 1-2 mm in diameter without angiogenesis providing abundant oxygen and nutrition. Since Folkman et al. proposed the concept of anti-angiogenesis in 1970s, the strategy was gained to widely research and had been an important complementary therapy for ovarian cancer especially for advanced ovarian cancer. Numerous anti-angiogenic drugs had been used in both preclinical and clinical trail, whereas the clinical responses didn't conform with expectation [40, 41]. Accumulating evidences suggested that angiogenic inhibitors such as bavacizumab, sorafenib, and sunitinib had no inhibiting effect on VM formation or instead induced VM formation *in vitro* and *in vivo* [5, 41], indicating the existence of VM might be one of important factors for poor respond of anti-agiogenic drugs only targeting vascular endothelial cells [31]. Consequently, a promising method to block tumorous blood supply was to inhibit both endothelium-dependent angiogenesis and VM formation.

It was well known that synthetic cRGD peptides inhibited blood vessels formed by endothelial cells through imitating endogenous RGD sequence to competitive and specific binding with integrin α_v_β_3_. Exogenous RGD presented promise as anti-angiogenic agent for endothelial cells and had been widely employed to tumor imaging and drug targeting, while there was no report about inhibiting VM formation by exogenous RGD. In this study, we disclosed a novel mechanism of VM formation accelerated by uPA in ovarian cancer and uPA could be a potent therapeutic target for VM. Mi and his co-worker found that OPN containing RGD sequence was blocked to bind with mammary epithelial cancer cell-surface integrins by exogenous RGD resulting in the decreased uPA expression [27]. Thus, we subsequently verified whether exogenous cRGD could be an antagonist of VM formation by down-regulating uPA expression. In the three-dimensional cultures, we observed that vessel-like channels were distinctly decreased both in SKOV-3 and OVCAR-3 cells after treating with cRGD. The results of western blot exhibited that cRGD down-regulated uPA expression, and its downstream proteins produced similar change as the interference experiments. Thus, it was demonstrated cRGD had ability to inhibit VM formation by down-regulating uPA expression and reconfirmed the signal pathway of uPA involving in VM formation in ovarian cancer.

**Figure 8:**
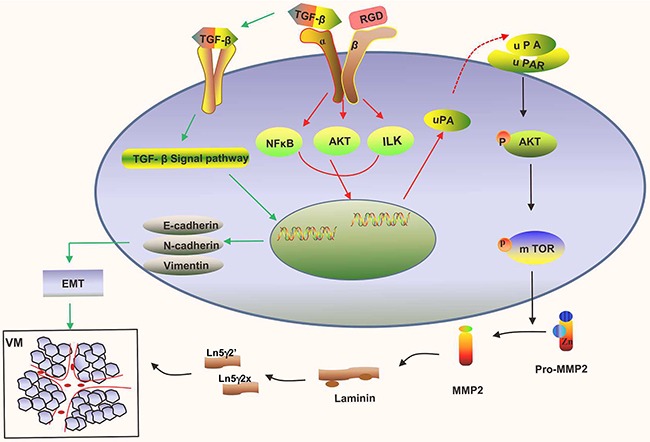
The diagram of uPA-mediated signal pathway for VM formation and the molecular mechanisms of cRGD inhibited VM formation in ovarian cancer

Integrins were critical intermediaries in the occurrence and development of malignant tumor. Reports suggested that LAPs of TGF-β contained RGD domain enabling to interact with integrin a_v_β_3_ resulting in TGF-β activation [42]. The process induced the occurrence of VM-featured EMT *via* TGF-β SMAD independent signaling [23]. In this study, we simultaneously detected cRGD could attenuate EMT in cell morphology and EMT-associated proteins including E-cadherin, N-cadherin, and Vimentin. In addition, it was found the redcuding EMT wasn't caused by uPA decrease. Therefore, our illustration demonstrated that cRGD inhibited VM formation in ovarian cancer *via* not only down-regulating uPA expression which reduced MMP-2 efficiency to cleave Laminin5γ2 through AKT/mTOR/MMP-2/Laminin5γ2 signal pathway but also reducing EMT (Figure 8).

Combined with our results, we first validated that uPA had important positive role on the VM formation by regulating AKT/mTOR/MMP-2/Laminin5γ2 signal pathway in ovarian cancer. Uncovering the relationship between uPA and VM provided a novel insight into the molecular mechanism of VM formation and seeked for a new VM antagonist for ovarian cancer. More importantly, our study demonstrated that exogenous cRGD had ability to inhibit VM by not only down-regulating the expression of uPA but aslo reducing EMT. Blocking the integrin α_v_β_3_ mediation by exogenous cRGD might be a promising strategies to thoroughly inhibit tumorous vessels for ovarian cancer due to cRGD effect on endothelial cells and VM channels.

## MATERIALS AND METHODS

### Cell culture and patient specimen collection

Human ovarian cancer cell lines SKOV-3, OVCAR-3, A2780 and Human endothelial cells HUVEC cells were purchased from Chinese Academy of Sciences (Shanghai, China). These cells cultured in DMEM (Corning, Virginia, USA) medium with 10 % fetal bovine serum and 0.1% penicillin and incubated at 37¼C in a humidified 5 % CO_2_ atmosphere. Ovarian cancer tissues were obtained from Department of Pathology, Zhujiang Hospital at Southern Medical University, Guangzhou, China. Tissue specimens were randomly collected from 90 untreated ovarian cancer patients before undergoing surgery between 2011 and 2014. The diagnoses of ovarian cancer tissues were verified by pathologistes. Tissue collection and analysis in this study were approved by the Research Ethics Committee of Southern Medical University (Guangzhou, China).

### MTT assay

SKOV-3 and OVCAR-3 cells were reseeded into 96-well culture plates at a density of 5 × 10^3^ cells/well and incubated at 37°C. After incubated with 24 h, cRGD with adaptive doses was added to plates and incubated with 48 h. And then the media were detached and replaced with 20 uL of MTT solution (5 mg/mL) followed by incubation for 4 h. After incubation, the solution was replaced with DMSO and the plates were slightly shaken. The plates were detected at 540 nm using a microplate reader (Biotek USA).

### Transient transfection with siRNAs

The siRNAs for uPA and negative control (si-NC) were designed and synthesized by Guangzhou RiboBio (RiboBio Inc, China). Target sequence of siRNA was: 5'-GAAGAGGUAUUGAAUGCUA-3'; anti-sense:3'-CUUCUCCAUAACUUACG AU-5'. uPA-specific and negative control siRNA were transfected into SKOV-3 and OVCAR-3 cells using Lipofectamine RNAiMAX Reagent (Life Technologies, Carlsbad, CA, USA) according to the manufacturer's protocol. Cells were then incubated with 48-72 h and collected to assess transfection efficiency by western blot and Real time-PCR and then were used in further experiments.

### Three-dimensional cultures and tubule formation

150 uL of Matrigel (BD Biosciences) was dropped on 24-well plates and incubated at 37°C for 45 min. SKOV-3, OVCAR-3 and HUVEC cells (3.5 × 10^5^ cells/well) were seeded into the surface of the gels and maintained in DMEM medium supplemented without serum to observe tubule structures formation, including the number and the completeness of the tubule. cRGD dissolved in PBS were added to cell suspensions before plating the cell into Matrigel using PBS as control. Images were captured using an inverted light microscope at 100x after incubating for 8 h.

### Migration and invasion assay

Cell migration assay was performed using Transwell cell culture inserts with 8 μm pores (Corning). Dissociated cells (1 × 10^5^/insert) in serum free medium were seeded on inserts and medium 10 % fetal calf serum was added to the lower chambers. After incubation for 12 h, the insert was gently washed with PBS, and the non-migrating cells on the upper membrane of insert were erased by cotton swab. The migration cells adhered to the membrane lower surface were fixed with cold 100 % formaldehyde for 10 min, stained with hematoxylin for 15 min and then number of cells was counted under a microscope in five random optical fields. For cell invasion assay, the matrigel was added to the inserts for 4 h before cells were plated into inserts.

### Western blot analysis

Western blotting analysis were performed with standard methods. Briefly, cell pallets of SKOV3 and OVCAR-3 cells were lysed in the RIPA buffer containing protease inhibitors and phosphatase inhibitors. Proteins were separated using 10 % SDS-PAGE gels, and then transferred to polyvinylidene difluoride membrane. After blocking in 3 % BSA for 1 h, the membranes were incubated with specific primary antibodies overnight at 4¼C, and then incubated with the secondary antibody (Zhongshan, Beijing, China) for 2 h at room temperature. GAPDH was used as a protein loading control. After repeatedly washed with TBST, the blots were enhanced by Western Blot chemiluminescence reagents Kit. The image were observed with ChemiDocTM CRS+ Molecular Imager (Bio-Rad, Hercules, CA, USA).

### Real time PCR analysis of uPA mRNA expression

The sequences of the primer for uPA was following: 5'- gggAATggTCACTTTTACCgAg-3', antisense primer: 5'-gggCATggTAgTTTgCTg-3'. Briefly, RNA was purified with TRIzol reagent (Invitrogen) from SKOV-3 and OVCAR-3 cells according to manufacturer's instructions. cDNA was synthesized with 1 μg RNA using the PrimeScript RT reagent Kit (TaKaRa). Quantiative PCR was conducted with SYBR Green dye at following cycle parameters: enzyme activation at 95¼C for 10 min, 40 cycle of 95 for 15 s, 60 for 30 s and 72 for 30 s. Data were normalized to GAPDH expression and all reactions were repeated triplicate using three independent synthesis of cDNA.

### Immunofluorescence

SKOV-3 and OVCAR-3 cells were seeded in the chamber slides in medium and incubated overnight at 37¼C. And then both cells were treated with cRGD for 48 h. The cells were washed twice with PBS, fixed with cold 4 % paraformaldehyde, permeabilized with 0.3 % Triton-X 100 for 15 min at room temperature and blocked with 5 % BSA for 30 min. Then the cells were incubated with E-cadherin antibody, N-cadherin antibody and Vimentin antibody overnight at 4¼C. Anti-rabbit IgG Alexa Fluor 488 were treated with cells for 1 h followed by Hochest33342 staining. The fluorescent images were viewed by confocal laser scanning microscope (Olympus FV1000, Japan).

### Immunohistochemistry and CD34-PAS double staining

Paraffin sections (5 mm) were deparaffinized using xylene and absoluted using ethanol, rinsed in PBS. Antigen retrieval was performed using citrate buffer (pH=6.0) for 5 min at 100¼C. After blocking with 10 % hydrogen peroxide, sections were incubated with rabbit polyclonal anti-CD34 and rabbit polyclonal anti-uPA overnight at 4°C. For CD34-PAS double staining, the paraffin sections was incubated with anti-CD34 followed by staining with PAS for 10 min. The section were counterstained with Hematoxylin for 5 min, and detected by a light microscope.

### Statistical analysis

SPSS (version 19.0; SPSS, Chicago, IL) was used for statistical analysis. All experiments were carried out at least in triplicate. All P-values were two sided, and < 0.05 was considered statistically significant.
